# Modified tibial cortex transverse transport for diabetic foot ulcers with Wagner grade ≥ II: a study of 98 patients

**DOI:** 10.3389/fendo.2024.1334414

**Published:** 2024-01-22

**Authors:** Junpeng Liu, Xingchen Yao, Ziyu Xu, Yue Wu, Fuchun Pei, Lin Zhang, Meng Li, Ming Shi, Xinru Du, Hui Zhao

**Affiliations:** ^1^ Department of Orthopaedic Surgery, Beijing Chaoyang Hospital, Capital Medical University, Beijing, China; ^2^ Department of Orthopaedic Surgery, Beijing Chaoyang Integrative Medicine Rescue and First Aid Hospital, Beijing, China

**Keywords:** complication, diabetes, diabetic foot ulcer, tibial cortex transverse transport, wound repair

## Abstract

**Background:**

Diabetic foot ulcers constitute a substantial healthcare burden on a global scale and present challenges in achieving healing. Our objective was to assess the efficacy of modified tibial cortex transverse transport surgery in managing refractory diabetic foot ulcers.

**Methods:**

We retrospectively analyzed clinical data from 98 patients suffering from diabetic foot ulcers classified as Wagner grade ≥II who were admitted to our medical facility between January 2020 and June 2022. All the patients were treated by modified tibial cortex transverse transport surgery, wherein the osteotomy scope was reduced to two rectangular bone windows measuring 1.5cm × 1.5cm each. Record the patient’s general information and ulcer healing time; ulcer area, ankle-brachial index, WIFi classification, and visual analogue scale before and 3 months following the surgical intervention.

**Results:**

The average duration of diabetes of 98 patients with diabetic foot ulcer was 20.22 ± 8.02 years, 52 patients had more than one toe gangrene on admission. The postoperative wound healing rate was 95.83% and the average healing time was 53.18 ± 20.18 days. The patients showed significant improvement in ankle-brachial index, WIFi classification, and visual analogue scale at 3 months postoperatively compared to preoperatively, with statistically significant differences (*P*< 0.05). Eight patients experienced complications, and the incidence of complications was 8.16%. Throughout the follow-up period, there were no instances of ulcer recurrence noted.

**Conclusion:**

Modified tibial cortex transverse transport surgery demonstrates effectiveness in the management of diabetic foot ulcers by enhancing lower limb microcirculation and facilitating the process of wound healing.

## Introduction

In 2021, type 2 diabetes was estimated to be 537 million worldwide (1), affecting one in every 11 adults (2). Diabetes and its complications pose a great threat to global health. Among them, approximately 19-34% of individuals with diabetes (3) will develop foot vasculopathy and neuropathy due to poor blood glucose control, leading to peripheral circulation blockage, foot deformities, and subsequent uneven pressure distribution on the feet. Coupled with diminished protective sensation, this cascade ultimately leads to skin ulceration, necrosis, and secondary infections. Simultaneously, due to compromised lower limb blood supply in patients and aggravated tissue damage from infections, this eventually leads to prolonged non-healing of diabetic foot ulcer (DFU) (4). Moreover, more than 15% of DFUs eventually necessitate amputation (3).

Although amputation surgery can enable patients to quickly return to normal life after surgery (5), it is associated with a high post-surgery recurrence rate, and the long-term prognosis is poor (6). Furthermore, the patients strongly desire limb preservation, making amputation surgeries difficult to carry out. The currently common clinical treatment methods include debridement, off-loading surgery, free flap transplantation, and vacuum sealing drainage. However, as these conventional methods often fail to enhance blood supply to lower limbs, patients’ ulcers often encounter challenges in achieving smooth healing. Although percutaneous transluminal angioplasty can restore patency in some major lower limb vessels, it cannot reconstruct the peripheral microcirculation, leading to limited effectiveness (7). At present, surgeons and DFU patients are faced with the problem that patients cannot achieve definite curative effects after paying huge medical expenses.

How to reconstruct or restore the blood perfusion to the distal limb is the key to the treatment of DFU. Wolff J (8) discovered that normal bone tissue possesses compensatory capability, allowing it to maintain dynamic metabolic equilibrium when subjected to external forces. Ilizarov G A (9) proposed the law of tension stress: giving suitable stretch stress to bone can stimulate the active growth of local tissues of the body, facilitate the restoration of microcirculation, subsequently augmenting local blood supply. Based on the above theory, Ilizarov G A designed and developed tibial cortex transverse transport (TTT) to address bone nonunion and found that TTT has the effect of promoting blood vessels and tissue regeneration (10). Subsequently, numerous researchers’ studies further affirmed the role of TTT in reconstructing local and distant tissue microcirculation and restoring blood supply (11). Hua et al. (12) took the lead in introducing TTT into the treatment of DFU, and reduced the corticotomy area of the original surgical method (5.0cm×1.5cm). Recent studies have shown that compared with conventional surgical treatment, TTT is more helpful for wound healing and limb salvage in DFU patients (13, 14). However, extensive osteotomy can result in excessive damage to the surrounding soft tissues, increasing the risk of bone flap necrosis and tibial fractures. Moreover, the angiogenic promotion effect does not intensify as the osteotomy area increases. In this study, to reduce the related complications caused by tibial osteotomy, we further reduced the osteotomy area by switching to two rectangular bone windows (1.5cm×1.5cm). The aim of this study is to assess the short-term effectiveness of modified TTT in managing DFU and to ascertain the occurrence of associated complications, thereby laying the groundwork for the surgical management of DFU.

## Methods

### Study population

This retrospective study used the clinical data of Wagner grade (15) ≥II DFU patients who underwent modified TTT at our medical facility between January 2020 and June 2022. Surgical indications (1): DFU with Wagner grade ≥II or showing no signs of remission for over 2 months following debridement, vacuum sealing drainage (VSD), and other traditional treatment methods; (2) participants aged 18 years or older; (3) ultrasonography or CT angiography showed that the popliteal artery and superficial femoral artery were unobstructed, with at least one branch of the posterior tibial artery, anterior tibial artery, and peroneal artery remained patent up to the ankle joint level. Surgical contraindications: (1) patients with life-threatening illnesses in the past 3 months or those who are currently unable to tolerate anesthesia and surgery; (2) patients diagnosed with tibial osteomyelitis; (3) the tibial incision area exhibited noticeable wounds, lesions, or infections within a 5cm distance from its edge; (4) disagreeing with the surgical treatment plan; and (5) individuals suffering from severe mental illness who were unable to engage in treatment collaboration. The selection of patients for TTT will be undertaken in accordance with the above. Patients were followed up for at least 3 months, excluding those lost to follow-up or with incomplete data.

### Preoperative preparation

Following hospital admission, the patients underwent detailed specialist assessment and routine preoperative examination. The abnormal blockage of the major supplying arteries (including popliteal artery anterior tibial artery, superficial femoral artery, peroneal artery, and posterior tibial artery) of the lower extremities was excluded by angiography or B-ultrasound examination of both lower extremities. Endocrine consultation was arranged, blood glucose was regularly monitored, and diet was controlled. The blood glucose control target was< 8.0mmol/L before meals and< 12.0mmol/L 2 hours after meals. Patients with infection and obvious foot ulcers were subjected to drug sensitivity test of bacterial culture medium of wound secretion, and anti-infective treatment was administered based on the drug sensitivity test results (16).

### Surgical technique

Modified TTT was performed using the following protocol: (1) The location of incision, corticotomy, and nail were marked before the operation. After the implementation of epidural anesthesia, the patient was positioned supine and subjected to standard disinfection procedures; (2) Starting at the upper third of the medial tibia (2cm below the tibial tubercle), two incisions of about 2cm in length were made along the medial side of the tibial diaphysis, with an incision interval of 3cm. The tissue was separated in layers until reaching the periosteum. The bone window locations were delineated on the medial aspect of the tibia, which were two 1.5cm×1.5cm rectangular bone windows. The periosteum was incised along the inner edge of the tibia and peeled off to both sides of the tibia. Then, the periosteum was lifted, and the periosteum integrity was protected during the whole process; (3) Two 3mm mobile external fixation pins were threaded into the bone windows (only screwed into the unilateral cortical bone) for transporting the bone blocks. Drill holes at the edge of the bone windows with a bone drill and connect the holes with a osteotome. The bone blocks were moved free to form mobile bone flaps. Damage to the marrow in the medullary cavity should be avoided during the operation; (4) Two 4mm external fixation needles were threaded into the proximal and distal tibial sides of the bone windows (penetrating both sides of the bone cortex). Install and tighten the external fixator. The subcutaneous skin and tissue were sutured in layers, and the incision was disinfected with 75% ethanol and bandaged with dressing. In cases of extensive wound infection or significant tissue necrosis, intraoperative debridement was executed. Pure dry gangrene cases did not necessitate specialized treatment.

### Postoperative management

**Figure 1:**
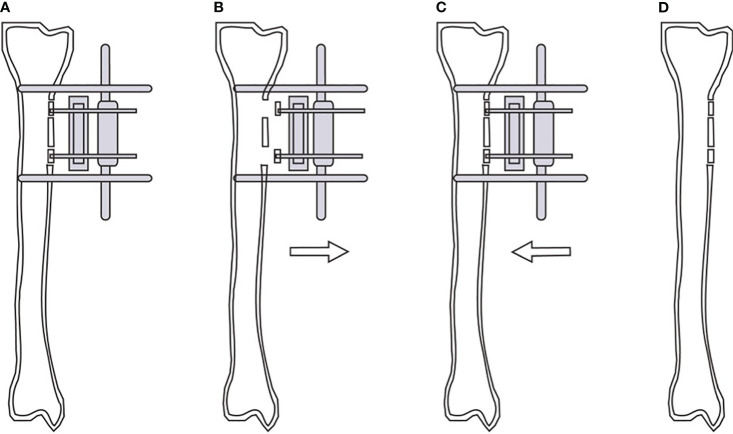
Schematic diagram of modified tibial cortex transverse transport. Position of bone windows opening, bone blocks fixation and external fixator screws during operation **(A)**. Continuous external traction of the bone blocks from the 5th day after operation **(B)**. Reverse traction of the bone blocks reduction after 2 weeks of traction **(C)**. Removal of the external fixator and gradual healing of the tibial cortex **(D)**.

Blood glucose was monitored regularly, and the blood glucose control range was< 8.0 mmol/L before meal and< 12.0 mmol/L 2 hours after meals (consistent with the preoperative management goal). The wound was bandaged with diluted iodine gauze, the wound dressing was changed every day and 75% alcohol was used to disinfect the needle site regularly. Antibiotics were routinely used to prevent infection and anticoagulants were given appropriately to prevent thrombosis. Cushion the heel and ankle with a soft pillow to reduce foot edema. Postoperative X-ray were obtained to determine the location of the incision and the placement of the screws for the external fixator (**Figure 1A**). External bone transport commenced on the fifth day post-surgery at a daily increment of 1 mm, divided into four sessions, spanning a duration of two weeks (**Figure 1B**), and then the radiographs were reviewed. After 3 days of maintenance, the bone was moved in the opposite direction at the same rate (**Figure 1C**). After 2 weeks, the tibial bone windows were moved back to the original position, and the X-rays were reviewed to evaluate the bone healing. When the wound showed complete epithelization without drainage and was maintained for 2 weeks, the ulcer was considered to be completely healed. Upon complete healing and stabilization of the bone blocks, the external fixator was removed (**Figure 1D**). Subsequently, a protective brace was applied to the operated side’s lower leg for a period of 6 to 8 weeks. And the patients with stable bone healing could walk normally.

### Assessment indicators

During the treatment, the relevant indicators of the patients were closely monitored. The clinical efficacy was evaluated by ulcer healing time, changes in ulcer area, wound/ischemia/foot infection classification (WIFi), ankle-brachial index (ABI) (17), and visual analogue scale (VAS) (18). Additionally, general patient information and adverse events were also documented. Assessors who were not part of the surgical procedure or routine wound care recorded indicators.

### Statistical analysis

Data were analyzed with IBM SPSS version 25.0 (IBM Corp, Armonk, NY, USA) and presented as $\left(\bar{x}\pm s\right)$ . Kolmogorov Smirnov test was used to determine the distribution form of preoperative and postoperative data. Paired-sample t-test was used to analyze the continuous measurement data with normal distribution. Wilcoxon rank-sum test was used for preoperative and postoperative non-parametric data or ranked data. *P<* 0.05 was considered statistically significant.

## Results

### General information and follow-up

Between January 2020 and July 2022, a total of 103 patients underwent modified TTT surgery. Five patients with incomplete data or loss of follow-up were excluded from this study. Ninety-eight patients were enrolled in the study (**Table 1**). Patients with toe gangrene before operation underwent toe amputation in the first stage. The wounds involved the toe in 77 cases, the foot in 33 cases, the heel in 2 cases, and the ankle in 3 cases. Ninety-eight patients were followed up for 3 months, which was terminated prematurely in cases of complete wound healing, wound expansion or patient mortality. Throughout the follow-up duration, 2 patients (2.04%) died due to severe perioperative complications (myocardial infarction/gastrointestinal bleeding, cerebral hemorrhage), thus, 96 patients were included in the follow-up statistics. The wound healing time was (21-108) days with an average of (53.18 ± 20.18) days.

**Table 1:** General information of DFU patients (n=98).

Characteristics	Total (n=98)
Age(years)	63.66 ± 13.02
Female, %(n)	35.71(35)
Poor blood sugar control, %(n)	69.39(68)
Course of disease(years)	20.22 ± 8.02
The time from diagnosis to surgery(days)	57.01 ± 85.86
Hypertension, %(n)	76.53(75)
Coronary herat disease, %(n)	30.61(30)
Number of gangrenous toes, %(n)	
≥3	5.10(5)
2	15.31 (15)
1	32.65 (32)
0	46.94 (46)

Data are presented as $\bar{x}\pm s$ or %(n).

### Clinical efficacy

**Figure 2:**
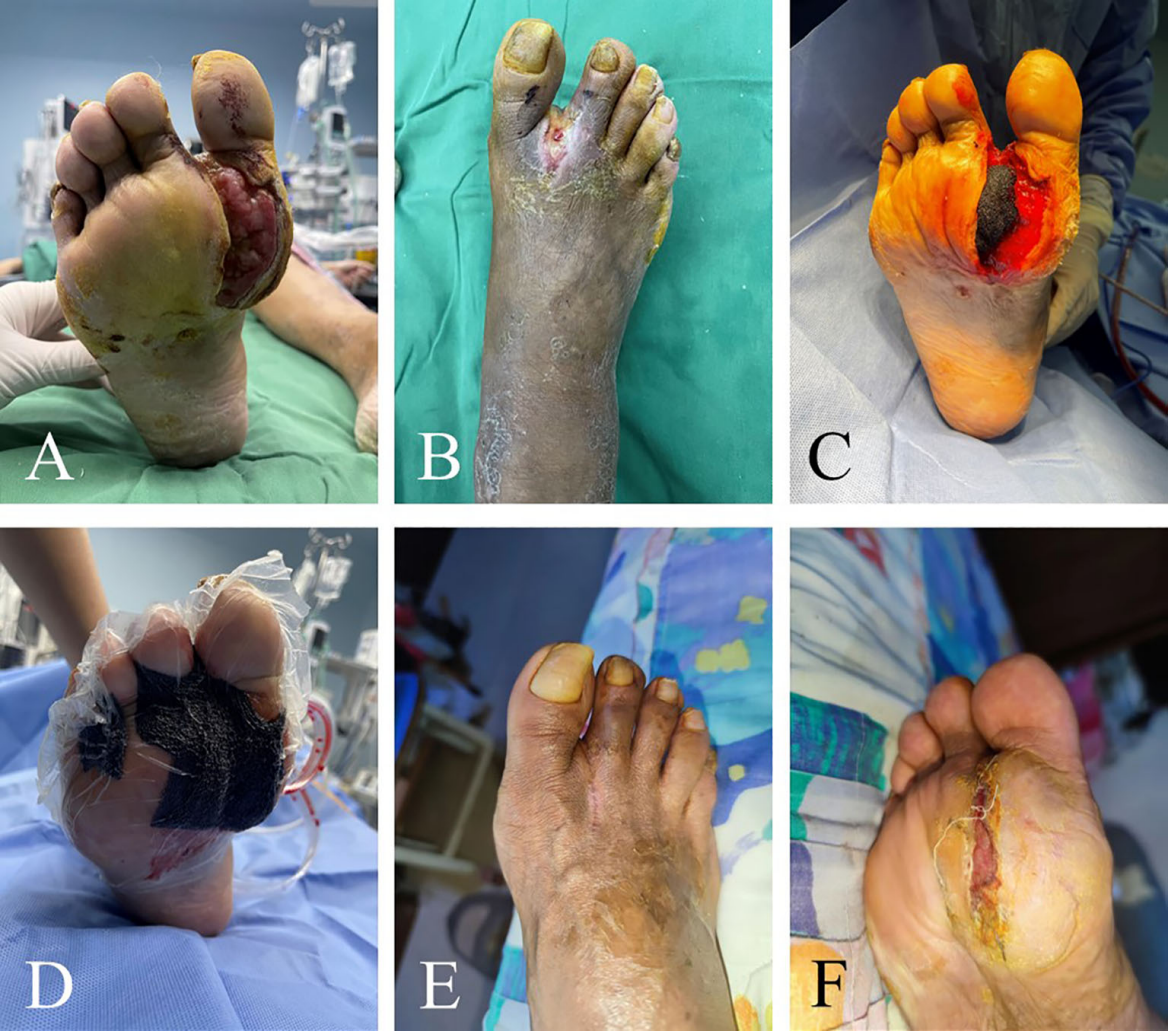
Efficacy of TTT for recalcitrant plantar ulcers. This 46-year-old male patient had skin ulcers on his right foot that lasted 6 months. He underwent conventional treatments such as debridement and lower extremity vascular intervention, but the progress of ulcers was not effectively curbed. These images show the distribution of ulcers in patients before surgery **(A, B)**. He had large ulcers on the right plantar thumb. There was purulent exudation at the web between the thumb and the second toe of the right foot. TTT was performed on the patient, and wound debridement and VSD placement were performed at the same time **(C, D)**. Ten weeks after surgery, the ulcers healed completely **(E, F)**.

**Figure 3:**
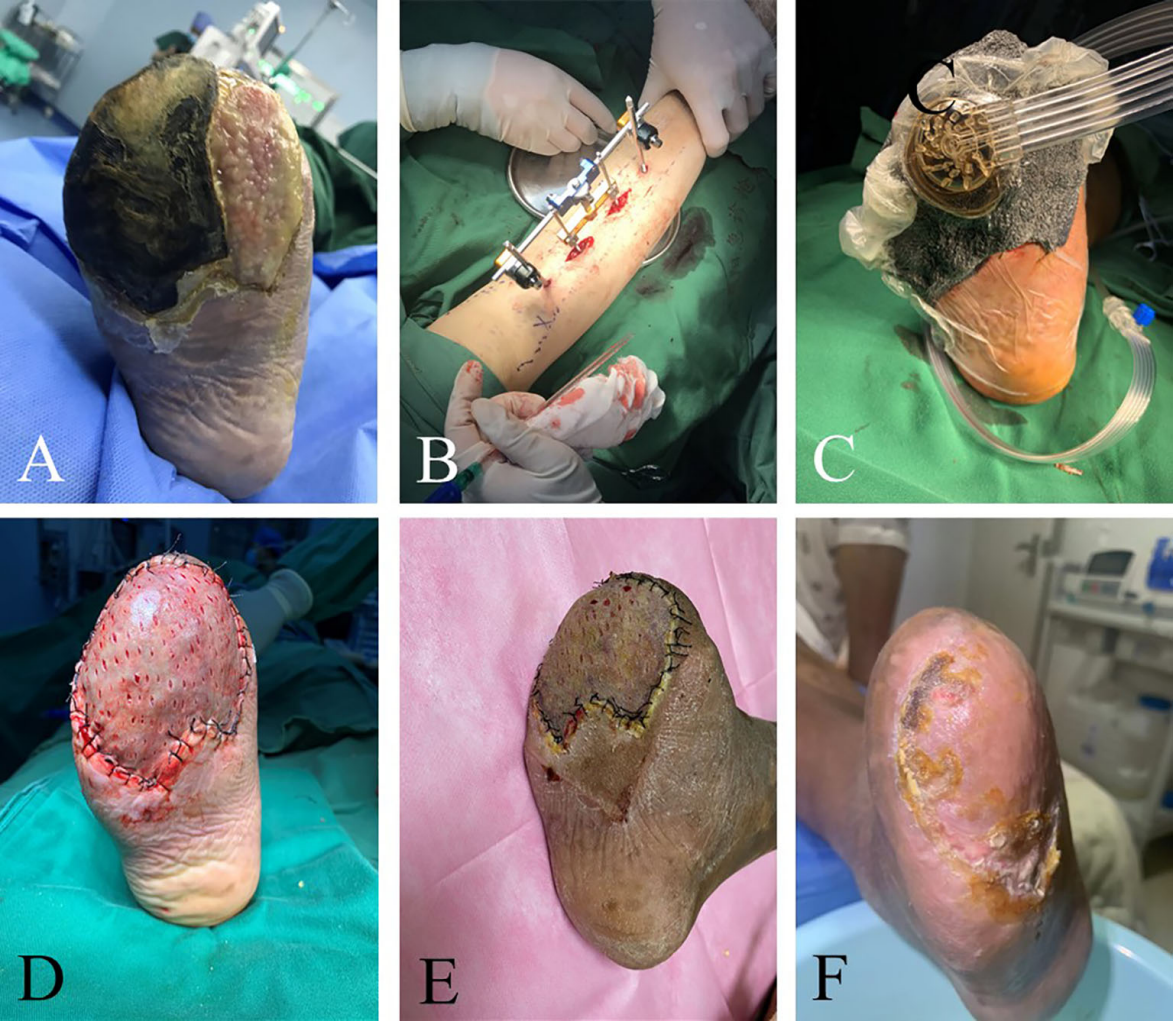
Efficacy of TTT in patients with recurrent ulcers after toe amputation and multiple debridement. The 41-year-old man’s right foot ulcers lasted for 3 months. He underwent multiple debridement, but the wound did not heal. Amputation was performed because of toe necrosis, and there were residual wounds after the operation. The image shows the patient’s ulcers before surgery **(A)**. There was purulent exudation on the surface of the ulcer and much necrotic tissue around it. Granulation tissue filling in the wound after right foot amputation. The swelling of the foot was evident. TTT was performed on patients with phase I wound debridement and VSD placement **(B, C)**. Thereafter, wound debridement and VSD replacement were performed weekly until the wound granulation tissue was completely fresh. In the third week after the operation, the skin grafting and the healing of the patients after skin grafting were observed **(D, E)**. There was epithelial formation at the edge of the wound. There was no pain, swelling, or infection in the wound. The ulcers healed completely 2 months after the operation **(F)**.

The WIFi classification of patients at 3 months after operation exhibited significant improvement compared to the pre-operative status, with the difference being statistically significant (*P*< 0.05, **Table 2**). At 3 months after operation, ABI was higher, wound area was significantly smaller and VAS was lower than that before operation. These differences were statistically significant (*P<* 0.05, **Table 3**). Ninety-two cases of foot wounds healed completely after operation and the healing rate was 95.83% (92/96) (**Figures 2**, **3**). In 4 cases of incomplete healing, the wound area was significantly reduced compared with that before operation.

**Table 2:** Comparison of preoperative and postoperative WIFi classification (n=96).

Period	W				I				Fi			
	0	1	2	3	0	1	2	3	0	1	2	3
Preoperative values(n)	0	2	71	23	0	42	47	7	0	1	83	12
Values at 3 months after operation(n)	92	4	0	0	32	63	1	0	96	0	0	0
*Z* value	-8.94				-7.47				-9.28			
*P* value	0.00				0.00				0.00			

W, wound; I, ischemia; Fi, foot infection.

**Table 3:** Comparison of preoperative and postoperative assessment indicators (n=96).

Characteristics	Preoperative values(n)	Values at 3 months after operation(n)	*Z* value or *t* value	*P* value
ABI	0.55 ± 0.03	0.66 ± 0.06	-18.39	0.00
Ulcer area(cm²)	13.17 ± 11.78	0.12 ± 0.52	-8.33	0.00
VAS	5.72 ± 1.17	0.79 ± 0.99	-8.56	0.00

Data are presented as $\bar{x}\pm s$ . The ABI data showed a normal distribution and was analyzed using a paired T-test; the remaining indices showed a non-normal distribution and were tested by Wilcoxon rank-sum test. ABI, ankle brachial index; VAS, visual analogue scale.

### Complications

**Figure 4:**
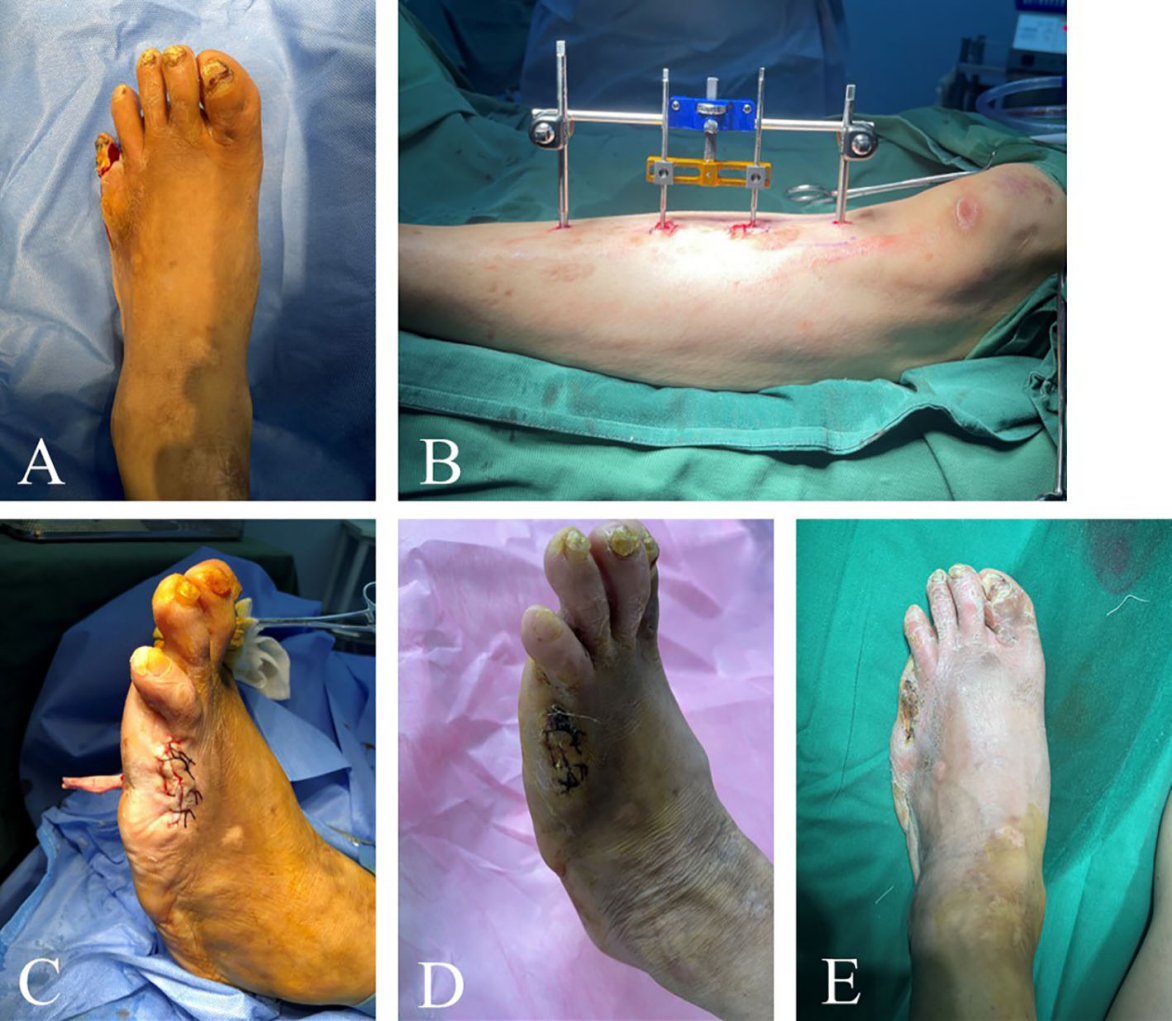
Efficacy of TTT in patients with multiple toe ulcers. This 68-year-old male patient had left foot bunion ulcers lasting 2 years and left little toe ulcers lasting 1 month. The patient changed dressing by himself several times, but the ulcers still recurred. Prior to surgery, the patient’s left foot was swollen and had pigmentation **(A)**. The skin of the little toe was ruptured with purulent secretion. The patient’s little toe was amputated and then TTT was performed **(B, C)**. Three weeks after surgery, the ulcer area was significantly reduced **(D)**. The wound at the toe amputation site was dry and healed well without infection. One month after surgery, the ulcers healed completely **(E)**.

A total of eight patients experienced complications and the overall complication rate was 8.16%. Among them, three patients underwent amputation following TTT because of uncontrolled ulcer infection, and the amputated incisions of these patients healed after receiving standard wound care (**Figure 4**). Two patients (2.04%) died during the follow-up period, with causes of death reported myocardial infarction/gastrointestinal bleeding and cerebral hemorrhage. Additionally, three patients developed pin tract infections after external fixation, and their infections were controlled successfully managed through alcohol irrigation of the pin tract and local disinfection. None of these patients progressed to osteomyelitis. There were no cases of ulcer recurrence or tibia fractures.

## Discussion

DFU is not only impose a significant financial burden due to the high treatment costs but also present a formidable challenge in terms of management, frequently culminating in non-traumatic amputations. At present, it is generally believed that diabetic foot ulcer is the joint result of vascular and neurological diseases of the lower limbs. Eighty percent of DFU patients undergoing amputation are related to foot ischemia (19), and small artery occlusion is the main cause of foot perfusion defects (20). Due to the poor vascular condition of lower limbs and the limited self-repair ability of the body, it is difficult to heal the wound by local debridement and dressing change alone. Studies have shown that after standard surgical treatment (21) (including decompression, infection control, revascularization, and wound debridement), the healing rate of ulcers within 1 year is 77%, and the recurrence rate are 40%, 60%, and 65% in 1, 3 and 5 years, respectively (22). It can be seen that although conventional surgery can heal the wound, the postoperative recurrence rate and amputation rate are still high due to the lack of intervention for the ischemic condition of the lower limbs of patients. Hence, the central challenge in the treatment of DFU lies in the restoration of blood supply to the distal foot, enhancement of microcirculation, and optimization of oxygen metabolism in neighboring tissues, all while conducting comprehensive debridement of infected tissue.

In the 1980s, Ilizarov introduced the Principle of Tension Stress: giving an appropriate stretch stress to the bone can stimulate the active growth of local tissues in the body, promote the rejuvenation of microcirculation and increase local blood supply. Subsequently, Ilizarov proposed TTT based on this theory and took the lead in applying this technique to the clinical treatment of bone nonunion, which achieved good therapeutic results. Later, Hua and his colleagues implemented this approach in the management of DFU and observed the postoperative ulcer healing rate was 96.3% and the 2-year recurrence rate was 2.9% (23). The therapeutic efficacy of TTT is evidently superior to conventional surgical treatment approaches. Further research found that TTT can improve the expression levels of angiopoiesis related factors and tissue repair-associated factors in the patients’ serum, consequently promoting angiogenesis (24–26). In addition, TTT also has a stimulating effect on immune cells. TTT has the ability to facilitate the transition of M1 macrophages into M2 macrophages during the wound healing proliferation phase, thereby promoting the polarization balance of macrophages and the reconstruction of anti-inflammatory function, and accelerating wound healing (27). Furthermore, VEGF-A released by M2 macrophages can also promote angiogenesis, guiding Schwann cells along neovascularization and inducing axonal regeneration, thereby improving patients’ neurological function (27). In this study, the osteotomy site was in the tibial diaphysis, but it was manifested as healing of foot ulcers (13, 14, 23, 28). This suggests that TTT can elicit a systemic response. This phenomenon may be related to the mobilization of bone marrow stem cells by TTT, which promotes the migration of stem cells to the site of injury, restores vascular endothelial function, and promotes angiogenesis (29). In addition, the occurrence of systemic reactions may be related to circulating cytokines produced by mechanical stimulation (30). TTT can provide effective pain relief. The decrease in VAS scores in patients after surgery is attributed to the reestablishment of collateral circulation in the foot and the reduction of intramedullary pressure through fenestration, which alleviates vascular spasms.

The modified TTT procedure used two small rectangular bone windows of 1.5cm×1.5cm, which reduced the overall bone window area. Small bone windows have significant advantages, including avoiding extensive periosteal stripping, reducing necrosis of free bone fragments, and maximizing the tibial stability and biomechanical integrity. The average incidence of postoperative tibial fractures using the traditional large area of osteotomy method was 2.6% (31). However, during the three-month follow-up period, no patient in our study experienced tibial fracture. Despite our follow-up period was relatively short, the complications related to TTT predominantly occur between 3 to 12 weeks postoperatively (31). Therefore, our record of complication rates remains valid. In comparison to the conventional osteotomy method, patients who underwent the modified TTT procedure had a significantly lower incidence of postoperative tibial fractures. This finding underscores the importance of reducing the dimensions of the osteotomy area is one of the key factors in preventing fractures, which is consistent with the views expressed by Wang et al. (31). It should be emphasized that the size of the tibial bone window is not entirely determined and can be appropriately reduced for patients with a shorter stature. In addition, due to the smaller osteotomy area, patients can engage in weight-bearing activities shortly after surgery, which serves to mitigate the risk of deep vein thrombosis formation in the lower limb. The postoperative ulcer healing rate in patients who underwent the modified TTT was 95.83%, and there was an increase in the ABI of the patients after treatment, consistent with findings from previous studies (13, 14, 23, 28). This indicates that reducing the size of the bone windows does not appear to weaken the subsequent traction’s stimulating effect on the periosteum, which is consistent with the findings of Kostopoulos et al.’s study (32). These results tentatively indicate the potential application of this technique for tissue regeneration therapy in smaller bone areas, such as the craniofacial region. However, further experimental studies are also necessary to validate these findings.

By transporting the bone blocks, the foot microcirculation and inflammatory balance were reconstructed. However, the efficacy of modified TTT should not be overstated. At the end of the follow-up of this study, there were still 4 patients whose wounds had not healed completely, and 3 patients underwent amputation due to the progression of local infection. And patients with DFU remain compromised static balance and alterations in plantar load distribution of the lower limbs following surgery. As can be seen from these adverse cases, DFUs are the result of multiple factors and cannot be expected to be completely cured by surgery alone. Thus, attention to patients’ self-management is crucial. Additionally, bone transport should be accompanied by thorough debridement, professional wound care, anti-infection treatment, static standing balance training, and blood glucose control.

It is worth noting that modified TTT is not a universal solution for all patients with DFU. Patients with mild ulcers often heal with conventional treatment and do not need modified TTT treatment. The effectiveness of TTT appears to be limited in individuals with severe or complete occlusion of the main vessels supplying the ankle. This limitation can be attributed to factors such as oxidative stress, impaired endothelial function, and regeneration defects commonly observed in diabetic patients. These factors collectively pose challenges for the regeneration and remodeling of large blood vessels through TTT, and the role of TTT in the reconstruction of middle and large vessels still requires further investigation.

The primary limitations of this study encompass the following aspects: Firstly, one major limitation of this study is the retrospective design of the study. Secondly, given the relatively recent emergence of this technique, our research was designed to evaluate the short-term therapeutic outcomes of modified TTT. In the future, we will conduct long-term large-sample controlled clinical trials on the basis of the original research to further clarify the therapeutic effect of modified TTT, and provide more reliable basis for limb salvage treatment of DFU.

## Conclusions

Modified TTT can effectively improve the foot microcirculation of patients, promote wound healing, and significantly reduce the amputation rate. After reducing the osteotomy area, the incidence of local tibial complications was reduced. In the future, more large-scale clinical trials are needed to further evaluate its efficacy. In addition, related experiments based on modified TTT are needed to explore its local biomechanical characteristics and related mechanisms.

## Data availability statement

The raw data supporting the conclusions of this article will be made available by the authors, without undue reservation.

## Ethics statement

The studies involving humans were approved by Beijing Chaoyang Hospital Human Research Ethics Committee. The studies were conducted in accordance with the local legislation and institutional requirements. The ethics committee/institutional review board waived the requirement of written informed consent for participation from the participants or the participants’ legal guardians/next of kin because Beijing Chaoyang Hospital Human Research Ethics Committee has confirmed that the need for consent to participate was deemed unnecessary according to national regulations and approved to access the patient date used in this research.

## Author contributions

JL: Conceptualization, Formal analysis, Investigation, Methodology, Writing – original draft. XY: Writing – original draft. ZX: Data curation, Writing – original draft. YW: Methodology, Writing – original draft. FP: Data curation, Writing – original draft. LZ: Data curation, Formal analysis, Writing – original draft. ML: Validation, Writing – original draft. MS: Formal analysis, Writing – original draft. XD: Methodology, Supervision, Writing – review & editing. HZ: Data curation, Supervision, Writing – review & editing.
